# Experimental investigation of water-saving efficiency using hexagonal diamond-shaped floating covers in large-scale evaporation ponds under static water conditions

**DOI:** 10.1371/journal.pone.0343523

**Published:** 2026-03-10

**Authors:** ZongLe Duan, KeBin Shi, KeWu Han, Tayi Abudula

**Affiliations:** 1 College of Hydraulic and Civil Engineering, Xinjiang Agricultural University, Urumqi, China; 2 Turpan Gaochang District Pucheng Water Conservancy Construction and Management Co., Ltd. Heigou Watershed Management Station, Turpan, Xinjiang, China; Universiti Malaysia Pahang Al-Sultan Abdullah, MALAYSIA

## Abstract

To investigate the evaporation suppression and water conservation effects of hexagonal diamond-shaped floating covers (HDFCs) under static water conditions, this study conducted experiments in circular evaporation ponds during the non-freezing period of 2024 (March 19 to November 30). The floating covers were arranged edge-to-edge and corner-to-corner, and daily water level observations were recorded. The evaporation reduction rate was calculated using the weighted average method. The results showed that with a porosity of 17.28%, the floating cover reduced evaporation by 1505.21 mm during the non-freezing period, corresponding to a water conservation rate of 74.86% relative to the total evaporation from the natural water surface (2010.7 mm). Theoretical analysis further indicated that if the floating covers fit perfectly with the pond edges, achieving a coverage rate of 98%, the theoretical water conservation rate could reach 88.59% based on the linear proportional scaling assumption. To validate practical applicability, supplementary experiments were conducted in a dynamic water environment (an unshielded large pond with natural wind and waves). The results demonstrated that the evaporation suppression rate remained above 70%, which is comparable to the results under static water conditions. This study confirms that hexagonal diamond-shaped floating covers exhibit effective evaporation suppression and water conservation performance under static water conditions and hold potential for practical application in open water bodies such as reservoirs.

## Introduction

As the global population continues to grow and irrigated agriculture expands, competition for increasingly scarce freshwater resources will intensify significantly [1]. This conflict is particularly acute in arid regions. Xinjiang, a typical representative of the arid regions in Northwest China, faces particularly severe water scarcity. The region exhibits distinct climatic characteristics, including large diurnal and seasonal temperature variations, low annual precipitation, frequent high winds, and extremely intense surface evaporation [2]. In Xinjiang’s plain reservoirs, evaporation accounts for over 40% of the total water volume [3]. Such substantial ineffective evaporation severely reduces the water resource utilization efficiency of plain reservoirs, ponds, and other water bodies. Against the backdrop of climate change and the greenhouse effect exacerbating the risk of aridification [4], water scarcity has become a key bottleneck constraining the sustainable social, economic, and ecological development of arid regions.

Scientifically reducing ineffective reservoir evaporation not only directly conserves precious water resources but also effectively mitigates the mineralisation concentration process in large water bodies caused by intense evaporation. Consequently, this lowers the associated risk of accelerated accumulation of secondary soil salinisation in irrigation districts. Thus, reducing evaporation losses from plain reservoirs is vital for promoting the efficient and sustainable use of water resources in arid regions and underpinning the stable socioeconomic development of these areas [5]. The substantial evaporative water loss significantly exacerbates low water resource utilisation efficiency, necessitating the implementation of measures to suppress surface evaporation.

Based on the existing literature, current mainstream research on surface evaporation suppression technologies can be broadly categorised into three groups: chemical reagent coverage, biological coverage, and physical material coverage. Barnes summarised laboratory experiments and findings on evaporation suppression using molecular films and discussed the challenges encountered in practical applications along with corresponding solutions [6]. Saggaï et al. conducted a 20-week experiment using three evaporation pans. Different doses of fatty alcohol were applied to different pans: the first pan was filled with water only (control), the second received fatty alcohol at the recommended concentration (0.3 kg per 10⁴ m² per day), and the third received a higher recommended concentration (0.5 kg per 10⁴ m² per day). Preliminary results indicated that the evaporation rate was reduced by 16% and 22% in the second and third pans, respectively, compared to the uncovered pan [7]. Mozafari et al. applied a chemical monolayer film to the water surface to suppress evaporation and investigated its mechanism. They found the monolayer was effective under conditions of no radiation and no wind. The primary mechanism identified for the reduced evaporation rate was the restriction of water molecule escape [8]. However, these films suffer from poor stability, low heat resistance, and are prone to accumulation and rupture under strong winds and wave conditions.

Biological coverage methods, such as floating plants, windbreaks, and palm leaves, can significantly reduce evaporation, but their use has limitations. Floating aquatic plants like water lilies (*Nymphaea*), duckweed (*Lemna minor*), giant duckweed (*Spirodela polyrhiza*), and watermeal (*Wolffia arrhiza*) can reduce reservoir evaporation by preventing connection at the air-water boundary layer. Studies in Thailand showed duckweed can reduce evaporation by up to 10%. However, not all aquatic plants are effective in lowering the evaporation rate. For instance, plants like lotus (*Nelumbo nucifera*) and water hyacinth (*Eichhornia crassipes*) have broad leaves that increase the transport surface area and consequently the evaporation rate [9].

The physical material coverage method involves using materials such as polystyrene foam boards, floating balls, and floating panels to form an air-isolating barrier on the water surface. This barrier provides shade, lowers water temperature, and effectively impedes water vapour transfer from the surface to the air. The core principle is to cover the water surface with materials possessing specific physical properties to avoid direct solar radiation and reduce direct contact between water molecules and air, thereby decreasing the evaporation rate. Under summer conditions, researchers measured hourly evaporation from two pans: one uncovered and the other covered with different types of shade nets. Nets tested included single-layer and double-layer polyethene nets in various colours, and a single-layer aluminised net. In all cases, the shade nets resulted in significant reductions in daily evaporation rates, ranging from 50% for the aluminised net to nearly 80% for the colored polyethylene nets [10]. Using natural physical material, palm leaves were employed as an evaporation suppressant. One pan was covered with a shade made of palm leaves bound with netting, while another remained uncovered. Results showed that an average of approximately 47% reduced evaporation from the covered pan compared to the uncovered pan. However, palm leaves have a short service life [11]. Jiang Haibo et al. employed polystyrene foam board (PS board) coverage technology to reduce ineffective evaporation in a plain reservoir. Outdoor experiments measuring evaporation reduction efficiency demonstrated a reduction in ineffective evaporation of 51.25%. Nevertheless, the fixing and connection methods for the boards require further optimisation [12]. Lehmann et al. used covers made of large 200 mm diameter discs, achieving an evaporation reduction rate of 80%, although the evaporation suppression tests were conducted only indoors [13]. The density of floating balls must be carefully considered, as using high-density solid balls can cause more than half of the ball volume to submerge, reducing coverage and potentially increasing evaporation [14]. In outdoor evaporation suppression experiments with polyethene (PE) floating balls, research on the effect of different ball colours found that black PE balls, due to their higher thermal conductivity, increased the temperature at the ball-air boundary layer, resulting in the lowest evaporation reduction rate [15]. Li Cunli et al. used floating panels and balls as primary coverage materials for evaporation suppression, conducting both laboratory and field tests. By adding weight to the balls, they solved the problem of irregular rotation and rolling under wind and wave action. Evaporation suppression rates for balls with diameters of 80 mm, 100 mm, and 120 mm were 39%, 45%, and 60%, respectively. They discovered that the suppression rate of floating panels was positively correlated with surface area and thickness. Ultimately, they concluded that floating balls are more suitable than panels for evaporation suppression in large reservoirs [16]. Assouline et al. proposed an open, lattice-like structure that suppresses evaporation while allowing light and oxygen transfer into the water. Laboratory experiments showed that the floating lattice structure reduced wind speed above the free water surface and suppressed evaporation by 40%−60%. However, the study was conducted under laboratory conditions, limiting the range of environmental factors considered [17]. Mady et al. conducted a multi-season study using eight identical evaporation ponds (13.5 m² each). Six ponds were covered (91% coverage) with white and black ethylene-vinyl acetate (EVA) foam discs (200 mm diameter, 15 mm thickness), leaving two uncovered. Results indicated evaporation suppression rates between 65% and 80% under summer field conditions [18]. Benjaoran et al. proposed reusing common polyethene terephthalate (PET) drinking water bottle caps to cover the water surface. Experiments using eight simulated ponds showed that among different opaque materials, PET bottles containing laminated aluminium foil (LAF) plastic bags provided the maximum evaporation reduction [19]. In outdoor evaporator coverage tests with expanded polystyrene (EPS) floating balls, it was found that water covered by 40 mm diameter EPS balls exhibited the highest average temperature and the largest relative humidity difference between the atmosphere and the space body. These balls achieved the highest evaporation reduction rate during the non-freezing period, reaching 76.31%, demonstrating optimal suppression effectiveness, though the study focused only on evaporation reduction in static water [20]. The role of floating high-density polyethene (HDPE) spheres in reducing reservoir evaporation and lowering water mineralisation was evaluated through laboratory and field tests assessing the effects of different coverage levels (0%−74.98%) on evaporation rate, sediment resuspension, and salinity. Results showed that covering 74.98% of the reservoir surface led to a 28.97% reduction in salinity over one irrigation cycle. Evaporation suppression varied from 13.56% to 60.19%, The dissolved oxygen concentration decreased by 1.21% to 8.54%, depending on the coverage level [21].

Reviewing the current status of research on evaporation suppression under physical material coverage, various structured physical covers have been investigated domestically and internationally in recent years to explore their effectiveness. When floating balls are closely packed, they exhibit a minimum porosity of at least 9% (The theoretical minimum porosity for the closest packing of floating spheres is approximately 9% [13,22]). However, hexagonal-diamond-shaped floats arranged edge-to-edge and corner-to-corner achieve a porosity significantly lower than 9%. Similarly, floating panels closely packed also have porosity less than 9%, but they lack practical fixing and connection methods. Under wind and wave conditions, gaps between panels can form or widen, causing the effective porosity to increase dramatically and leading to a sharp decline in water-saving efficiency.

Compared to the aforementioned solutions, the Hexagonal Diamond-shaped Floating Covers (HDFCs) possess dual advantages in terms of geometry and mechanical performance:

(1) Very Low Theoretical Porosity: Their unique hexagonal tiling structure allows for tightly packed, edge-to-edge and corner-to-corner arrangements, achieving a porosity significantly lower than that of the closest packing of spheres (theoretically below 5%). This maximizes the coverage of the water surface, directly blocking the primary pathways for water vapor escape.
(2) Excellent Structural Integrity and Wave Resistance: A key design feature is a one-way conical vent valve at the base of each unit. This valve allows water to slowly enter the internal cavity under wave action, forming ballast that is difficult to expel quickly. This design naturally lowers the center of gravity and enhances stability, significantly improving resistance to wind and waves without altering the external geometry. Furthermore, the units can be integrated via reliable connection methods into a large-area, monolithic cover layer that is rigid or semi-rigid. This integrated structure effectively resists displacement, rolling, and stacking induced by wind and waves, maintaining a long-term stable state of low porosity. This overcomes the inherent drawbacks of spherical floats being prone to rolling and floating panels being prone to stacking, constituting a critical engineering feature for achieving sustained and highly efficient evaporation suppression.

Despite these advantages, their effectiveness in evaporation suppression and water conservation remains unclear. This study aims to quantify the evaporation reduction rate of hexagonal-diamond-shaped floats through field experiments in static water, clarify the water-saving patterns across different periods, and provide a novel solution for evaporation control in plain reservoirs within arid regions.

## Materials and methods

### Basic information of the test site

The experiment was conducted in Kunyu City, Hotan Prefecture, Xinjiang Uygur Autonomous Region, China, in a temperate arid desert climate zone characterized by distinct seasons, hot summers, and cold winters. Meteorological data indicate the following climatic parameters (long-term averages):

Annual mean temperature of 12.2°C, with extreme maxima/minima of 40.6°C and −21.6°C, respectively. Monthly averages range from −5.6°C (coldest month) to 25.5°C (hottest month). Diurnal temperature variations exceed 15°C, particularly during spring and autumn. Annual precipitation averages 33.4 mm, contrasting sharply with an evaporation potential of 2,620 mm, reflecting severe aridity. Annual sunshine duration reaches 2,769.5 hours (62% sunshine rate). The mean wind speed is 3.4 m/s, predominantly from western and northwestern directions, with maximum gusts of 16 m/s. The region experiences frequent aeolian activity between April and June: > 200 floating dust days and 18–52 sandstorm days annually. Frost-free 244 days, maximum frozen soil depth of 0.67 m, and average annual occurrence of 11.5 gale events.

The study area exemplifies hyperarid conditions, marked by minimal humidity, intense evaporation, frequent windborne particulates, and abundant solar energy resources. These climatic features underscore the critical need for evaporation suppression technologies in regional water resource management.

### Selection of materials

Based on prior research, effective evaporation suppression materials must exhibit optimal density, high mechanical strength, hydrophobicity, non-toxicity, environmental adaptability, cost-effectiveness, water quality neutrality, and long-term durability. The hexagonal diamond-shaped floating covers (HDFCs) employed in this study not only meet these criteria but also incorporate two innovative features:

(1) Submerged Conical Stabilization: Each HDFC unit features a hollow lower cone with water intake ports. During deployment, these cones fill with water, significantly increasing self-weight to resist wind displacement under non-extreme wind speeds (<15 m/s).
(2) Interlocking Geometric Configuration: Edge-to-edge and corner-to-corner alignment minimizes inter-unit porosity while enhancing structural coherence under hydrodynamic stresses (e.g., waves, currents).

The HDFCs were fabricated from high-density polyethylene (HDPE) through rotational molding, achieving a hollow monolithic structure with wall thickness uniformity (±0.5 mm). polyethylene plastic has proved to be the most acceptable and durable material for covers of this type [23]. High-density polyethylene (HDPE) is an ideal material to use, since it is widely used in hydraulic engineering and aquaculture industries and is unlikely to cause water quality problems. this material is unlikely to cause problems associated with the progressive leaching of chemicals from the spheres into the water, and it is able to resist UV radiation [2,22].

**Fig 1:**
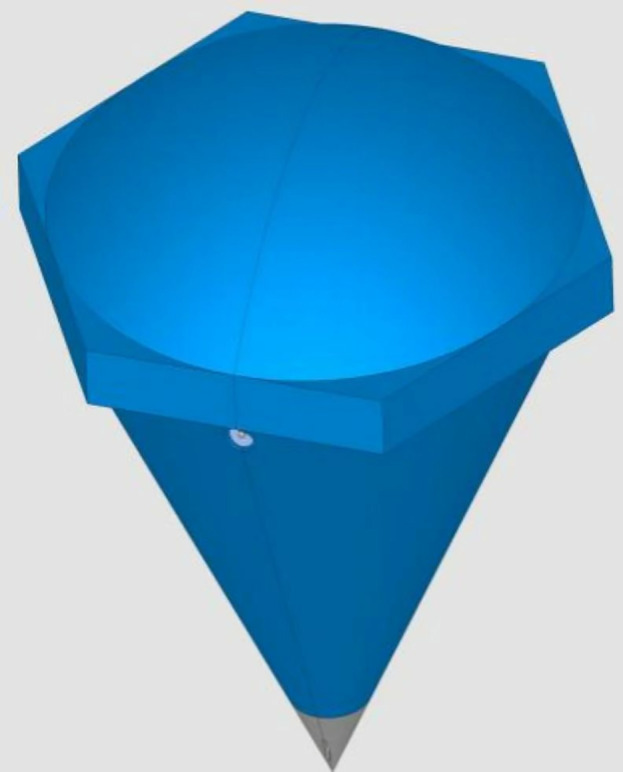
Schematic diagram of the hexagonal diamond-shaped floating covers.

**Fig 2:**
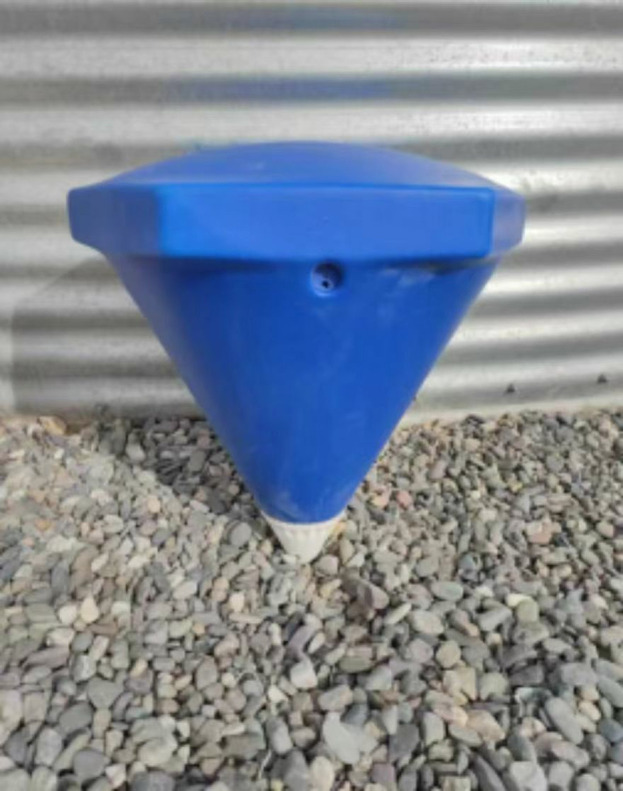
Real photo of the hexagonal diamond-shaped floating covers.

Full specifications are detailed in Table 1, with structural schematics in Fig 1 and field deployment images in Fig 2.

**Table 1:** The main parameters of the hexagonal diamond-shaped floating covers.

Material	Structure	Component parts	Area of each covered water area (m^2^)	Cost (CNY per piece)
High-density polyethylene	Integral type, wall thickness 2 mm, side length 22.5 cm, height 42 cm	Floating integral, one-way conical exhaust valve	0.1315	23

The hexagonal diamond-shaped floating covers were fabricated using high-density polyethylene (HDPE) with a material density of 0.955 g/cm³. To enhance resistance to capsizing and displacement under wave conditions, a one-way conical vent valve was integrated into the base of each floating unit. The design principle of this valve allows water to enter the internal cavity of the unit unidirectionally under wave-induced pressure fluctuations while preventing its rapid outflow. The water entering the cavity acts as an adjustable internal ballast, effectively lowering the center of gravity of the floating unit and increasing its righting moment. Consequently, this design significantly improves the hydrodynamic stability of the unit without altering its external geometry.

### Test principle

Evaporation, a phase transition from the liquid to the vapor state driven by solar energy absorption, is governed by temperature, humidity, wind velocity, and vapor pressure gradients—water molecules at the surface gain kinetic energy through solar radiation, enabling their escape into the atmosphere. The evaporation rate correlates positively with water surface temperature, air-water humidity differentials, and wind-induced turbulence.

Hexagonal diamond-shaped floating covers (HDFCs) suppress evaporation through three synergistic mechanisms:

(1) HDFCs exhibit high albedo properties, reflecting a significant portion of incident solar radiation, thereby reducing heat absorption by the underlying water body. This lowers the water surface temperature, decreasing molecular kinetic energy and evaporation rates.
(2) The impermeable structure of HDFCs acts as a thermal insulation barrier, decoupling heat transfer between the atmosphere and water. By physically blocking vapor diffusion, HDFCs reduce the effective water-air interface area (>95% coverage). A near-saturation humidity microenvironment forms beneath the cover, minimizing vapor pressure gradients and suppressing evaporation.
(3) The HDFCs array suppresses wind speed effects through its covering layer, effectively reducing wind velocity near the water surface and diminishing the air flow’s capacity to transport water vapor. The closely arranged hexagonal diamond configuration attenuates wind-induced disturbances on the water surface, thereby decreasing evaporation rates. Furthermore, humidity accumulation beneath the coverage elevates local humidity levels, which reduces the water-air humidity gradient and consequently inhibits evaporation through thermodynamic regulation.

In conclusion, the hexagonal diamond-shaped floating structure mitigates water evaporation through multiple synergistic mechanisms, including:

(1) reduction of water temperature through thermal regulation.
(2) obstruction of vapor diffusion pathways.
(3) suppression of wind-induced surface turbulence.
(4) minimization of exposed evaporation surface area.

The efficacy of these structures is contingent upon three critical parameters: the photothermal properties of constituent materials, spatial coverage integrity across the water-air interface, and environmental adaptability to varying meteorological conditions.

### Calculation of coverage area and porosity

The porosity (P) of the cover layer is defined as the ratio of the uncovered water surface area to the total pond surface area. This value was derived geometrically as follows:

The circular evaporation pond had a diameter *D* = 6, giving a total water surface area *So*:


$$S_{o}\;=\;{\pi \;(D/2)}^{\text{2}}\;\approx \;28.27\;m^{\text{2}}\mathrm{\backslash ]}$$

A single regular hexagonal floating unit had a side length (a = 0.225 m). Its projected area *S*_*A*_ was calculated as:


$$S_{A}=\frac{3\sqrt{\text{3}}}{\text{2}}\times a^{\text{2}}\approx \;0.1314\;m^{\text{2}}\;(using\;\sqrt{\text{3}}\;\approx \;1.732)\mathrm{\backslash ]}$$

The total number of complete units deployed was *K* = 178. Thus, the total covered area *S*_*cover*_ was:


$$S_{cover}\;=\;K\;\times \;SA\;\approx \;23.3892\;m^{\text{2}}.\mathrm{\backslash ]}$$

The coverage ratio *C* and porosity *P* were then derived:


$$C\;=\;S_{cover}/{\;S}_{o}\;\approx \;82.72\%,\;P\;=\;1\;-\;C\;=17.28\%.$$

P = 1 − C =17.28%

Regarding spatial variation, throughout the experimental period under static water conditions, the floating units maintained a stable, edge-to-edge and corner-to-corner configuration without stacking, tilting, or significant displacement. Therefore, the covered area remained constant, and no appreciable variation in porosity across the pond surface was observed.

### Test layout

This experimental study investigates large-scale evaporation ponds, which include circular evaporation ponds under static water conditions and large evaporation ponds in dynamic water environments.

#### Experimental setup for static water conditions.

The experiment was conducted at an open, well-ventilated site adjacent to a reservoir, where two identical circular evaporation ponds (Pond A and Pond B) were installed. Each pond measured 6 m in diameter and 2.3 m in height, with a water surface area of 28.27 m². Both ponds’ inner walls and bases were treated with waterproofing and thermal insulation to minimize lateral heat transfer and seepage. Due to the wind-shielding effect of the circle pond walls, the side wall is higher than the water surface. When the measured wind speed about 5 cm above the side wall is 4.8m/s, the wind speed about 5 cm above the water surface drops to 0.0m/s. Therefore, it can be regarded as an evaporation test under still water conditions. Due to the confinement of the evaporation pond’s vertical sidewalls, the hexagonal units are deliberately arranged in a tightly interlocked configuration, edge-to-edge and corner-to-corner. Even when subjected to wind forces, the overall coverage layer experiences minimal deformation and displacement, maintaining constant coverage. Consequently, the impact on experimental results is negligible.

**Fig 3:**
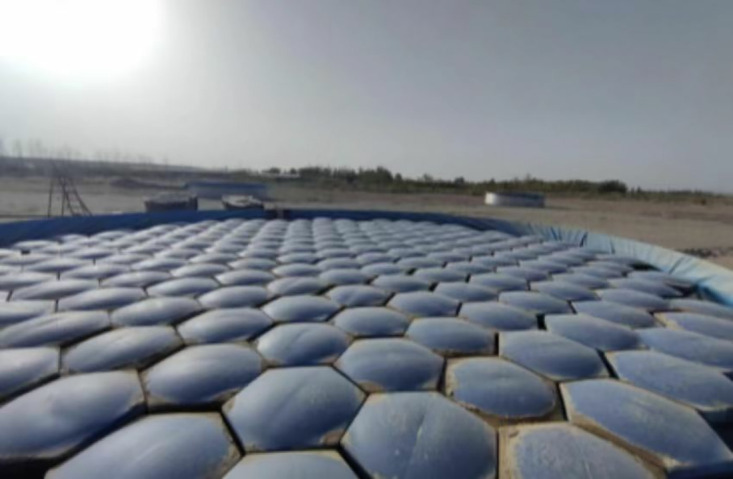
Group A Circular evaporation pool.

**Fig 4:**
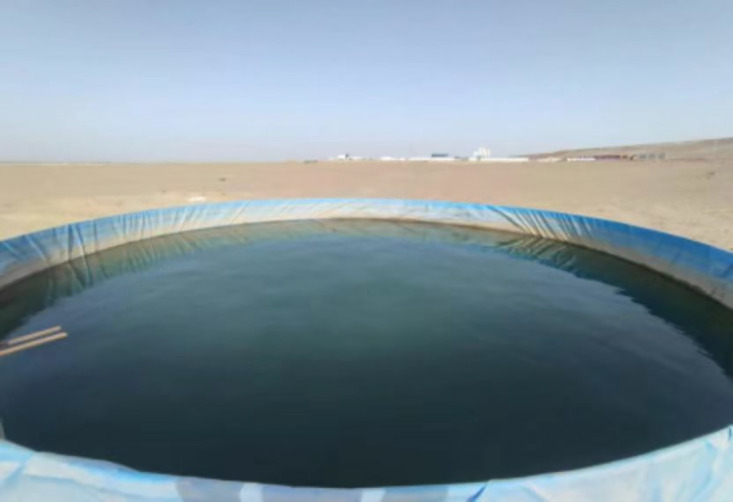
Group B Circular evaporation pool.

Pond A was entirely covered with hexagonal diamond-shaped floating covers (HDFCs, Fig 3), arranged in edge-to-edge and corner-to-corner configurations. However, geometric constraints imposed by the circular pond walls limited the final coverage to 82.72%. Pond B served as an uncovered control Fig 4). Water level variations in both ponds were monitored daily at 20:00 using calibrated rulers with a precision of 0.1 mm. The circular evaporation ponds were replenished to their respective initial water levels every three days. The replenishment water was sourced locally, with its temperature closely matched to that of the pond water. Before replenishment, the water temperature was measured to ensure a temperature difference within 1°C, thereby minimizing thermal disturbance to the pond’s heat balance during the refilling process.

An automatic weather station was deployed adjacent to the ponds to continuously record atmospheric temperature, wind speed, humidity, and barometric pressure. The site requirements and layout scheme of the evaporation ponds fully comply with the provisions of Section 2.2.2 in 《SL630–2013 Standard for Water Surface Evaporation Observation》: the distance between two evaporation pans shall not be less than 4 m, and the water surface area of each evaporation pan after being fully filled shall be greater than or equal to 1 m². An NK5500 meteorological station was installed at a height of 1.5 m above the ground in the experimental site, and key meteorological parameters including wind speed, wind direction, air temperature, relative humidity, and atmospheric pressure were automatically collected and recorded at 10-minute intervals.

#### Experimental setup for dynamic water conditions.

**Fig 5:**
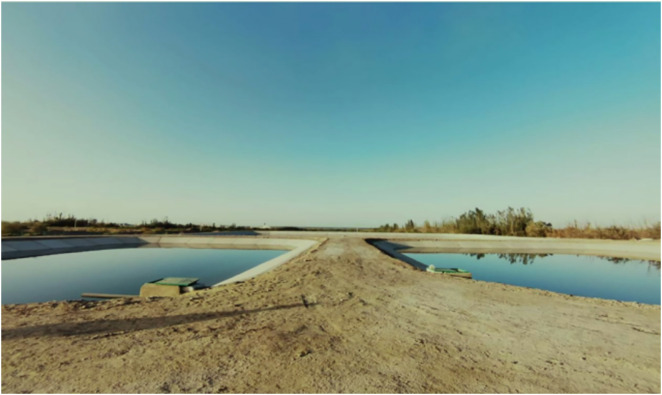
Large evaporation Pond 1 (right) and Pond 2 (left).

A dynamic water test site was established in an open, well-ventilated area near a reservoir. Two large evaporation ponds, designated as Pond 1 (1055 m²) and Pond 2 (884 m²), were constructed and put into operation in early October 2023 (Fig 5). Water level gauges were installed along the pond banks, and daily readings were taken at 20:00 to determine evaporation based on water level changes.

**Fig 6:**
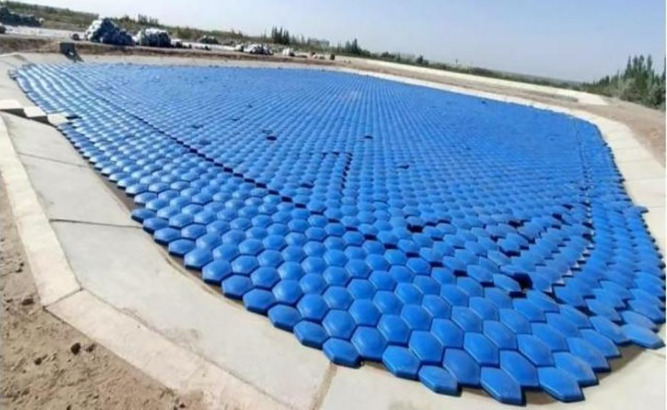
Large evaporation Pond 1.

To evaluate the performance of hexagonal diamond-shaped floating covers (HDFCs) under dynamic water conditions, a supplementary experiment was conducted from June to September 2024. During this period, HDFCs were deployed on Pond 1 (1055 m²). This pond was located in an unsheltered area where wind-induced wave action was evident, thereby simulating the dynamic environment typical of actual reservoirs (Fig 6). Water level changes continued to be monitored daily using the same method described above for evaporation calculation.

## Results and analysis

### Observed evaporation reduction rate

In this study, water level fluctuations within the circular evaporation ponds are exclusively attributed to evapotranspiration, as the pond’s interior walls and base are fully sealed. The evaporation reduction rate (*e*) within the interstices of the hexagonal diamond-shaped floating covers (HDFCs) is defined as:


$$e=\frac{E_{1}-E_{2}}{E_{1}}\times 100\%$$ (1)

*e*=evaporation reduction rate (%);

*E*_*1*_ = evaporation from the natural water surface (mm);

*E*_*2*_ = evaporation from the HDFC-covered water surface (mm).

The experiment was conducted non-freezing from 19 March to 30 November 2024. Water level variations were monitored under two conditions: uncovered and HDFC-covered ponds. Data were systematically recorded and subjected to statistical analysis.

Table 2 presents the measured evaporation volumes during different time intervals and the daily average evaporation volumes for the circular evaporation ponds tanks of Group A and Group B during the non-freezing period.

**Table 2:** Evaporation amount and daily evaporation rate for each time period during the non-freezing period in the two circular evaporation ponds.

Date (Month/Day)	Evaporation volume (mm) of HDFCs laid in the circular evaporation pond of Group A	Evaporation volume (mm) of the circular evaporation pond in Group B without any cover	Daily Evaporation Rate of group A (mm/day)	Daily Evaporation Rate of group B(mm/day)
3/19-3/31	14.9	70.5	1.15	5.42
4/1-4/30	47.1	200.7	1.57	6.69
5/1-5/31	74.2	306.2	2.39	9.88
6/1-6/30	80.2	314.3	2.67	10.48
7/1-7/31	88	346.8	2.84	11.19
8/1-8/31	68.2	268.3	2.2	8.65
9/1-9/30	53.2	205.9	1.77	6.86
10/1-10/31	45.7	173	1.47	5.58
11/1-11/30	32.9	125	1.1	4.17
3/19-11/30	504.4	2010.7		

As shown in Table 2, the total evaporation from the circular evaporation ponds covered with hexagonal diamond-shaped floating covers (Group A) under static water conditions was 504.4 mm during the experimental period (19 March to 30 November). These findings underscore the potential for significant evaporation reduction in circular evaporation ponds.

The evaporation reduction rate is influenced by meteorological conditions (e.g., temperature, humidity), leading to significant variations in evaporation across different periods. The simple arithmetic mean assumes equal contributions from all periods, failing to capture the differences in suppression effectiveness under varying weather conditions. In contrast, the weighted average, based on actual evaporation volumes, better aligns with the physical process. By assigning greater weight to periods with intense evaporation, it more accurately represents the actual evaporation suppression performance of the covering measure during peak water consumption periods.

We utilized the weighted average evaporation reduction rate calculation to ensure a robust analysis. This method assigns weights proportional to the evaporation contribution of each sub-period relative to the total experimental duration. By giving more weight to periods with higher evaporation, the algorithm ensures a more accurate representation of temporal variability in suppression efficiency. The weighting factor for each sub-period is defined by Equation (2):


$${\text{w}}_{\text{n}}=\frac{E_{\text{n}}}{E_{\text{a}}}\times 100\%$$ (2)

where:

*w*_*n*_= weighting factor for the n-th time period, %;

*E*_*n*_= evaporation amount during the n-th time period, mm;

*E*_*a*_ = cumulative evaporation from the first to the n-th time period, mm.

The weighted average evaporation reduction rate is calculated using the following general formula (3):


$$e_{a}=\frac{w_{\text{1}}*e_{\text{1}}+w_{\text{2}}*e_{\text{2}}+\cdots +w_{\text{n}}*e_{\text{n}}}{w_{\text{1}}+w_{\text{2}}+\cdots +w_{\text{n}}}$$ (3)

Where:

*e*_*a*_: Weighted average evaporation reduction rate (%)

*w*_*1*_,*w*_*2*_,…,*w*_*n*_: Weighting coefficients for each time interval (dimensionless), representing the proportion of evaporation in each interval relative to the total evaporation over the study period

*e*_*1*_,*e*_*2*_,…,*e*_*n*_: Evaporation reduction rates (mm) measured in the evaporation pond during the 1st, 2nd,..., n-th time intervals

Since the weighting coefficients are defined as the ratio of each interval’s evaporation to the total evaporation (*w*_*1*_ + *w*_*2*_ +  ⋯ + *w*_*n*_ = 1), Equation (3) reduces to:


$$e_{a}=w_{\text{1}}*e_{\text{1}}+w_{\text{2}}*e_{\text{2}}+\cdots +w_{\text{n}}*e_{\text{n}}$$ (4)

Equation (4) is mathematically expressed as: The weighted average evaporation reduction rate (WARR) over the entire study period is calculated by summing the products of the time-specific weights and their corresponding evaporation reduction rates.

The weights of each period in Group A were determined by dividing the evaporation amount of individual periods by the total evaporation amount (504.4 mm) from 19 March to 30 November, as described in Equation (2). These ratios represent the proportional contribution of each period to the total evaporation in the evaporation pool of Group A.

According to Formula (1), the evaporation reduction rates under the coverage of HDFCs. at each period can be calculated, as shown in Table 3 The weights and evaporation reduction rates of each period are shown in in Table 3.

**Table 3:** The weights of each period in the evaporation pool of Group A and the evaporation reduction rate of each period.

Date (Month/Day)	Weight w (%)	Evaporation reduction rate e (%) under hexagonal diamond-shaped float covers
3/19-3/31	2.95	78.87
4/1-4/30	9.34	76.53
5/1-5/31	14.71	75.77
6/1-6/30	15.90	74.48
7/1-7/31	17.45	74.63
8/1-8/31	13.52	74.58
9/1-9/30	10.55	74.16
10/1-10/31	9.06	73.58
11/1-11/30	6.52	73.68
3/19–11/30 (Total)	100.00	

**Fig 7:**
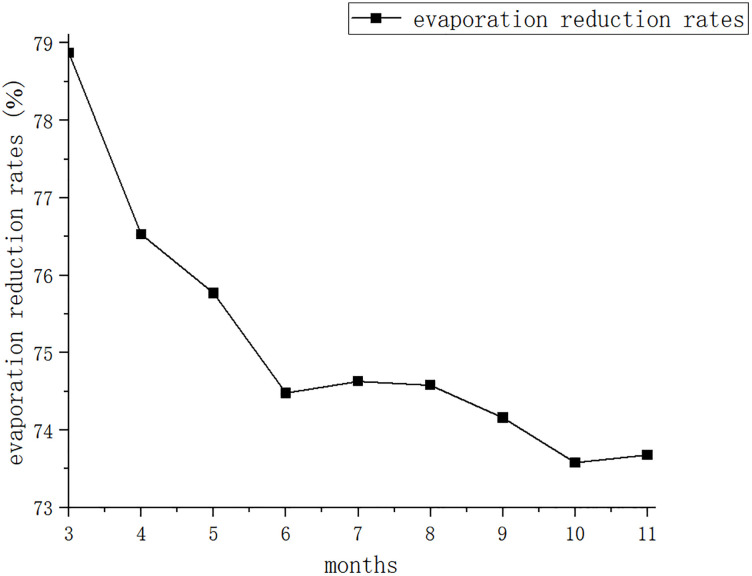
A line graph of the evaporation reduction rate of a circular evaporation pool covered by a hexagonal diamond-shaped floating covers in each month. Note: March only includes the period from 19 March o 31 March.

Fig 7 was generated based on the evaporation reduction rates of hexagonal diamond-shaped floating covers across different periods listed in Table 3.

As shown in Fig 7, the evaporation reduction rates exhibit a general decreasing trend throughout the observed periods.

**Fig 8:**
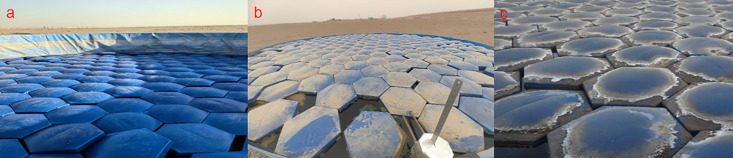
Operational status and dust accumulation on hexagonal diamond-shaped floating covers at different times: (a) 6 January 2024; (b) 14 April 2024; (c) 20 November 2024.

Fig 8 illustrate the long-term performance of hexagonal diamond-shaped floating covers (HDFCs). A comparison across the three figures indicates that the local climate is characterized by frequent wind and dust. The edges of the hexagonal diamond-shaped floater became covered with accumulated dust over long-term operation. Due to capillary action, these edges remained consistently moist. Over time, as dust accumulation increased, the moistened area expanded. This phenomenon likely contributes to the overall decreasing trend observed in the evaporation reduction rate from 19 March to 30 November.

Evaporation volumes during different time intervals and daily evaporation volumes of the two circular evaporation ponds tanks were summarized in Table 4.

**Table 4:** Average air temperature, relative humidity, and corresponding evaporation reduction rate for each monitoring period during the non-freezing season.

Date (Month/Day)	Mean air temperature (℃)	Mean relative humidity (%)	Evaporation reduction rate e (%) under HDFCs
3/19-3/31	16.02	22.86	78.87
4/1-4/30	19.11	22.33	76.53
5/1-5/31	25.36	24.22	75.77
6/1-6/30	26.71	33.42	74.48
7/1-7/31	28.56	36.84	74.63
8/1-8/31	27.48	42.02	74.58
9/1-9/30	22.45	41.83	74.16
10/1-10/31	16.09	32.97	73.58
11/1-11/30	8.02	42.53	73.68

#### Statistical analysis of temporal trends in evaporation reduction.

The monthly evaporation reduction rate (e) demonstrated a discernible declining trend over the experimental period (Table 4). Linear regression confirmed a statistically significant negative trend with a slope of −0.645% per month (R_2_ = 0.825, p = 0.0006). The non-parametric Mann-Kendall test corroborated the presence of a significant monotonic decreasing trend (p = 0.001).

To attribute this trend, a multiple linear regression was performed with e as the dependent variable and time (month sequence as a proxy for dust accumulation), mean monthly air temperature, and mean relative humidity as independent variables. The analysis revealed that time was the only statistically significant predictor of the decline in e (standardized coefficient β = −0.876, p = 0.003). Neither temperature (p = 0.710) nor humidity (p = 0.630) showed a significant independent effect, strongly supporting the hypothesis that progressive dust accumulation and consequent surface wetting were the dominant drivers of the observed performance attenuation.

To attribute this trend, a multiple linear regression was performed with *e* as the dependent variable and time (month sequence as a proxy for dust accumulation), mean monthly air temperature, and mean relative humidity as independent variables. The analysis revealed that time was the only statistically significant predictor of the decline in e (standardized coefficient β = −0.876, p = 0.003). Neither temperature (p = 0.710) nor humidity (p = 0.630) showed a significant independent effect, strongly supporting the hypothesis that progressive dust accumulation and consequent surface wetting were the dominant drivers of the observed

Using the proportional contribution of evaporation in each sub-period (relative to the total evaporation from 19 March to 30 November) as temporal weights (Table 3), we calculated the weighted average evaporation reduction rate for HDFC-covered periods. These weighted values, derived by multiplying sub-period evaporation reduction rates by their corresponding weights, are systematically presented in Table 5.

**Table 5:** Weighted evaporation reduction rates in each period.

Date (Month/Day)	Weighted evaporation reduction rate in each period (%)
3/19-3/31	2.33
4/1-4/30	7.15
5/1-5/31	11.15
6/1-6/30	11.84
7/1-7/31	13.02
8/1-8/31	10.08
9/1-9/30	7.82
10/1-10/31	6.67
11/1-11/30	4.81
3/19–11/30 (Total)	74.86

Table 5 shows that the (weighted) average evaporation reduction rate from 19 March to 30 November during the non-freezing period is 74.86%.

### Theoretical evaporation reduction rate

During the deployment of hexagonal diamond-shaped floating covers (HDFCs) in this experiment, the vertical sidewalls of the 6-m-diameter evaporation pond constrained the arrangement, resulting in an actual coverage rate of 82.72%. Notably, if HDFCs with curved edges matching the pond’s boundary were utilized, the theoretical coverage could reach 98%. The ratio of actual-to-theoretical coverage corresponds to the evaporation reduction rate under 82.72% coverage relative to the theoretical rate under 98% coverage for each observation period. This relationship is quantified by Equation (5):


$$\frac{82.72\%}{98\%}=\frac{e_{e}}{e_{t}}$$ (5)

Based on the assumption of linear proportional scaling, the measured efficiency at 82.72% coverage was extrapolated to the theoretical efficiency at 98% coverage. After multiplication and division transformation of Formula (5), Formula (6) can be obtained:


$$e_{t}=118.47\%e_{e}$$ (6)

In this context, *e*_*t*_ represents the theoretical evaporation reduction rate at 98% coverage, while *e*_*e*_ denotes the evaporation reduction rate at 82.72% coverage. Using Equation (1), the theoretical evaporation reduction rates for the HDFCs system in the circular evaporation ponds were calculated for each time interval during the non-freezing period (19 March to 30 November). Multiplying these theoretical reduction rates by the evaporation values of Group B yielded the corresponding theoretical evaporation amounts under 98% coverage. Detailed results are summarized in Table 6.

**Table 6:** The theoretical evaporation reduction rate and theoretical evaporation volume in each period.

Date (Month/Day)	Theoretical evaporation reduction rate (%) in each period	Theoretical evaporation (mm)
3/19-3/31	93.43	4.63
4/1-4/30	90.67	18.73
5/1-5/31	89.76	31.35
6/1-6/30	88.24	36.96
7/1-7/31	88.41	40.20
8/1-8/31	88.36	31.24
9/1-9/30	87.86	25.00
10/1-10/31	87.17	22.19
11/1-11/30	87.29	15.89
3/19–11/30 (Total)		226.19

As shown in Table 6, the evaporation reduction rates exhibit a general decreasing trend throughout the observed periods.

According to Equation (4), using the ratio of theoretical evaporation in each period to the total theoretical evaporation at 98% coverage as a weighting factor, the weighted theoretical evaporation abatement rate for each period was calculated by multiplying these weights by their corresponding theoretical evaporation abatement rates. The weighted average evaporation abatement rate from 19 March to 30 November was subsequently determined by summing all weighted theoretical evaporation values across the periods. The specific weights and weighted theoretical evaporation rates for each period are presented in Table 7.

**Table 7:** The weights and weighted theoretical evaporation rates of each period.

Date (Month/Day)	Weight (%)	Weighted theoretical evaporation rate (%)
3/19-3/31	2.05	1.91
4/1-4/30	8.28	7.51
5/1-5/31	13.86	12.44
6/1-6/30	16.34	14.42
7/1-7/31	17.77	15.71
8/1-8/31	13.81	12.20
9/1-9/30	11.05	9.71
10/1-10/31	9.81	8.55
11/1-11/30	7.02	6.13
3/19–11/30 (Total)	100.00	88.59

It can be seen from Table 7 that the theoretical average evaporation reduction rate of the non-freezing period under hydrostatic conditions, calculated by weighted average, is 88.59%.

### Water-saving volume and efficiency

Under the uncovered condition of a large evaporation pond, the total evaporation (*E*_*₀*_) during the non-freezing period (19 March to 30 November) was measured as 2010.7 mm. The amount of evaporation prevented during this period was calculated by multiplying the total evaporation by the weighted average evaporation reduction rate from 19 March to 30 November. The computational results are presented in Table 8.

**Table 8:** Evaporation prevention during the test period of the hexagonal diamond-shaped floating covers (Unit: mm).

The total amount of evaporation prevention during the non-freezing period	1505.27
The total evaporation of natural water surfaces during the non-freezing period	2010.7

The total amount of anti-evaporation during the non-freezing period is 1505.27 mm. Under still water conditions, the anti-evaporation water-saving rate of the circular evaporation pool covered by hexagonal diamond-shaped floating covers during the non-freezing period is 74.86%.

### Evaporation reduction rate during the freezing period and year-round water conservation potential

Although this study focused on the non-freezing period, the year-round performance and practical implementation considerations of the HDFC covers warrant further discussion.

During the freezing period, the evaporation suppression mechanism transitions from blocking the loss of liquid water to inhibiting ice sublimation and sub-ice vapor diffusion. Given that the covers exhibit a constant porosity (17.28%), the proportion of exposed ice surface area remains consistent with this porosity fraction. The covers achieve sublimation inhibition through two primary mechanisms: first, acting as a physical barrier at the ice–atmosphere interface, and second, providing a thermal insulation effect that dampens the temperature gradients driving vapor diffusion. Based on this, the theoretical sublimation inhibition rate can approximate the coverage rate (≈82.72%).

Total sublimation during the freezing period can be approximated as being solely contributed by the exposed ice surfaces within the porous regions, whereas sublimation in the covered regions is significantly suppressed. If residual diffusion in the covered regions is neglected, the theoretical sublimation inhibition rate during the freezing period can reach approximately 82.72%. Evaporation during the freezing period is substantially lower than that during the non-freezing period. Based on the calculation method for the weighted average evaporation inhibition rate, the annual evaporation reduction rate is slightly greater than 74.86%.

This estimation indicates that the hexagonal floating covers exhibit sustained and high-efficiency water conservation potential on an annual scale.

### Experimental study of HDFCs on evaporation ponds under dynamic water conditions

As the water level gauges in the large evaporation ponds could only measure the total water level change, which is the sum of evaporation and seepage losses, it was necessary to quantify the seepage component. The average seepage rate under steady-state conditions was adopted for this purpose.

**Fig 9:**
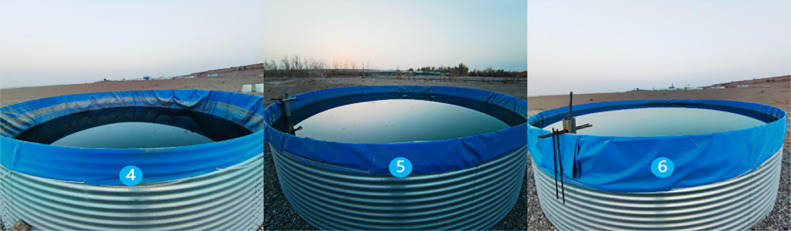
Circular Evaporation Ponds 4, 5, and 6 during the uncovered period.

The daily water level changes in the blank control evaporation ponds (Ponds 4, 5, and 6, each 6 m in diameter, see Fig 9) and the blank Ponds 1 and 2 were measured, yielding the data presented in Tables 9 and 10.

**Table 9:** Daily Water Level Changes in Blank Control Circular Evaporation Ponds and Large Evaporation Ponds 1-2 in November 2023 (Unit: mm).

Date (Month/Day)	Water Level Change in Evaporation Pond 4	Water Level Change in Evaporation Pond 5	Water Level Change in Evaporation Pond 6	Water Level Change in Large evaporatio-n Pond 1	Water Level Change in Large evaporation Pond 2
11/1	9.2	9.7	8.3	15.6	11.6
11/2	4.6	5.4	4.8	13.0	8.0
11/3	4.5	5.4	5.1	13.1	8.0
11/4	4.8	5.3	4.9	13.3	8.1
11/5	4.7	4.7	4.7	12.4	7.6
11/6	4.4	4.3	4.3	11.4	7.4
11/7	4.8	4.9	4.6	11.2	7.2
11/8	3.6	3.6	3.6	10.5	6.5
11/9	3.5	3.9	3.7	10.6	6.5
11/10	4.9	4.4	4.8	11.1	7.1
11/11	2.9	3.2	3.0	9.5	6.7
11/12	3.9	4.2	4.1	9.6	6.6
11/13	2.4	2.9	2.6	9.6	6.6
11/14	3.2	3.6	3.4	10.1	6.0
11/15	3.1	3.7	3.3	10.0	6.0
11/16	3.1	3.7	3.1	10.2	6.1
11/17	3.0	3.1	3.4	8.6	5.6
11/18	2.2	2.1	2.1	9.1	5.0
11/19	2.8	2.8	2.4	9.2	5.1
11/20	2.2	2.2	2.2	8.9	4.8
11/21	3.0	2.6	2.9	9.8	5.7
11/22	2.2	3.2	2.6	9.3	5.2
11/23	2.8	3.0	2.9	9.5	5.3
Total	85.8	92	86.8	245.6	152.7
Average	3.7	4.0	3.8	10.68	6.64

**Table 10:** Daily Water Level Changes in Blank Control Circular Evaporation Ponds and Large Evaporation Ponds 1-2 in December 2023 (Unit: mm).

Date (Month/Day)	Water Level Change in Evaporation Pond 4	Water Level Change in Evaporation Pond 5	Water Level Change in Evaporation Pond 6	Water Level Change in Large Evaporation Pond 1	Water Level Change in Large Evaporation Pond 2
12/1	1.9	2	2.2	4.8	3.7
12/2	1.9	2.1	2.2	4.7	3.7
12/3	1.6	1.6	1.6	4.6	3.4
12/4	2	1.9	1.7	4.8	3.7
12/5	2.6	2.7	2.6	5.1	4.0
12/6	2.5	2.7	3.6	5.4	4.3
12/7	2.5	2.5	2.2	5.2	4.0
12/8	2.4	2.4	2.2	5.0	3.9
12/9	2.1	2.1	2	4.7	3.6
12/10	2.7	3	3.7	5.4	4.3
12/11	2.7	3	3.6	5.2	4.1
Total	24.9	26	27.6	54.9	42.7
Average	2.26	2.36	2.51	4.99	3.88

In Table 9 the average of the mean daily evaporation from Ponds 4, 5, and 6 (each 6 m in diameter) was calculated as (3.7 + 4.0 + 3.8)/ 3 = 3.8 mm. Using 3.8 mm as the estimated mean daily evaporation for Ponds 1 and 2, the mean daily seepage for Pond 1 was derived as 10.68 - 3.8 = 6.88 mm, and for Pond 2 as 6.64 - 3.8 = 2.84 mm, based on the measured mean daily water level changes from November 1–23.

Similarly, from Table 10, the average mean daily evaporation for the control ponds was (2.26 + 2.36 + 2.51)/ 3 = 2.38 mm. Applying this value, the mean daily seepage for Pond 1 was calculated as 4.99 - 2.38 = 2.61 mm, and for Pond 2 as 3.88 - 2.38 = 1.51 mm, based on data from December 1–11. The seepage rate stabilized, and the mean daily seepage rate for Pond 1 in 2024 was therefore taken as 2.61 mm/d.

The seepage loss and the measured water level change for Pond 1 from June to September 2024 are summarized in Table 11 below. The evaporation from the pond was calculated as the water level change minus the seepage loss.

**Table 11:** Presents the seepage loss and water level changes for the large Pond 1 from June to September 2024.

Date (Month)	Water Level Change in Large Evaporation Pond 1 (mm)	Seepage Loss (mm)	Evaporation from Large Evaporation Pond 1 (mm)
6	171.1	78.3	92.8
7	181	80.91	100.1
8	159.8	80.91	78.9
9	140.1	78.3	61.8

As Circular Pond 4 (Group B Circular evaporation pool) was covered with HDFCs during the non-freezing period of 2024, the average evaporation from the blank control Circular Ponds 5 and 6 during June-September 2024 was used to represent the evaporation from the natural water surface of Pond 1. Combined with the evaporation data for the large pond from Table 11, the evaporation reduction rate was calculated using Equation (1), yielding the results in Table 12.

**Table 12:** Evaporation Reduction Rate for Large Pond 1 from June to September 2024.

Date (Month)	Evaporation from the Natural Water Surface of Large Evaporation Pond 1 (mm)	Evaporation Reduction Rate (%)
6	317.5	70.77
7	350.3	71.42
8	270.2	70.80
9	209.2	70.46

As shown in the table above, the 1055 m² pond covered with hexagonal diamond-shaped floating bodies (HDFCs) under dynamic water conditions affected by wind and waves maintained an evaporation reduction rate above 70%. This result is comparable to the findings from static condition experiments conducted during the same period, demonstrating that the core evaporation suppression efficacy of the HDFCs is not significantly compromised by wind-induced waves.

### Water footprint and life cycle assessment

#### Water footprint analysis.

The manufacturing of hexagonal diamond-shaped floating covers itself carries a water footprint. Producing each kilogram of the cover material requires 0.11 m³ of water. After covering the circular evaporation pond in Group B with these floating covers, the evaporation during an entire non-freezing period decreased by 1506.3 mm. With the evaporation pond having a radius of three meters, this translates to water savings of approximately 42.59 m³ per non-freezing period. A total of 178 floating covers were used in the circular evaporation pond, each weighing 1.8 kg, requiring 35.24 m³ of water to manufacture. The production-related water consumption can be “repaid” after only 35.24/ 42.59 ≈ 0.83 non-freezing periods (about 7 months) of operation. All water savings beyond this period represent net gains. This demonstrates that the environmental “water benefit” of this technology is rapid and significant.

#### Life cycle carbon footprint assessment.

Energy consumption and carbon footprint during the manufacturing stage: Based on life cycle inventory data, producing 1 kg of polyethylene consumes approximately 78 MJ of primary energy and emits about 1.8 kg of CO₂ equivalent. The total mass of floating covers used in this experiment is 320.4 kg, resulting in an embodied carbon footprint of approximately 577 kg of CO₂ equivalent for the manufacturing stage.

Carbon emission reduction benefits from water savings: To comprehensively assess environmental benefits, the aforementioned carbon cost was linked to the water-saving benefits during the operational phase. Assuming the floating covers operate effectively for 20 years, the estimated total water savings based on the annual water-conservation data from this experiment is approximately 852 m³. In arid regions, alternative water supply solutions such as long-distance water transfer or seawater desalination typically have high carbon intensities. For example, the energy-related carbon intensity of water conveyance for China’s South‑to‑North Water Diversion Project (Middle Route) is about 0.34 kg CO₂ equivalent/m³, while the carbon intensity of many seawater desalination projects can reach 1.0–2.0 kg CO₂ equivalent/m³. Using a conservative estimate (0.5 kg CO₂ equivalent/m³) for calculation, the water-saving achieved by this floating cover array avoids approximately 426 kg of CO₂ equivalent in water-supply-related emissions. This result indicates that, considering only the indirect carbon emission reduction from water savings, it is expected to offset most (about 74%) of the initial manufacturing carbon cost over the typical lifespan of the project, highlighting the environmental sustainability potential of this technology on a full life‑cycle scale.

End-of-Life Disposal and Recycling Potential: High-Density Polyethylene (HDPE) is highly recyclable. After the floating covers reach the end of their service life, a mechanical recycling process is recommended. This process includes collection, cleaning, shredding, melting, and pelletizing, ultimately allowing the material to be reprocessed into plastic products with lower performance requirements, such as municipal infrastructure or packaging pallets. Recycling HDPE can save approximately 70–90% of the energy compared to producing virgin material and proportionally reduce associated carbon emissions and water consumption, thereby significantly lowering the overall life cycle carbon footprint of this technology. Therefore, establishing a well-developed recycling system is a critical step in enhancing its environmental sustainability.

### Microplastics impact and service life of floating covers

#### Microplastics impact.

Numerous studies have demonstrated the effectiveness of HDPE covering materials in water conservation. However, there is still a lack of empirical research on their long-term aging under strong ultraviolet radiation in arid regions and the release of microplastics. This represents a critica direction for future research in this field. Subsequent studies should systematically sample and monitor microplastics in the covering materials themselves, in the reservoir water and sediments, as well as in the soils of downstream irrigation areas.

#### Service life of floating covers.

The service life of the floating covers has been validated through long-term field applications. The HDPE-based floating covers developed by our research team were first deployed in 2015 at the Shengjingou Reservoir, located in the heart of the “Flaming Mountains” in Turpan, Xinjiang. They have since operated safely and stably for 10 years. To further verify their durability, a portion of these covers, which had already been in service for 8 years, was relocated to the experimental site in Kunyu City, Hotan Prefecture, in early 2023. They continue to function normally to date. Furthermore, the shell material of the hexagonal diamond-shaped floating covers incorporates appropriate amounts of UV absorbers, light stabilizers, and antioxidants during production. These additives effectively delay material aging under intense UV radiation and oxidative environments. Based on more than 10 years of actual operational records and the material’s weather-resistant design, the expected service life of these floating covers is estimated to reach 20 years.

### Statistical analysis

#### Error analysis.

Due to a water level gauge accuracy of ±0.1 mm, certain observational errors exist. The cumulative error from March 19 to November 30 was ± 25.7 mm. During the non-freezing period, the total evaporation from the circular evaporation pond covered with Group A hexagonal diamond-shaped floats was 504.4 ± 25.7 mm, while that from Group B circular evaporation pond was 2010.7 ± 25.7 mm. The arithmetic mean evaporation reduction rate was calculated as follows:(2010.7 - 504.4)/ 2010.7 × 100% = 74.91%. The maximum evaporation reduction rate was: [(2010.7 + 25.7) – (504.4 - 25.7)]/ (2010.7 + 25.7) × 100% = 76.49%, which is 1.58% higher than the arithmetic mean evaporation reduction rate. The minimum evaporation reduction rate was: [(2010.7 - 25.7) – (504.4 + 25.7)]/ (2010.7 - 25.7) × 100% = 73.29%, which is 1.62% lower than the arithmetic mean evaporation reduction rate.

Thus, the percentage error introduced by the observation ranged from −1.62% to +1.58%.

The error percentage in the calculated data was determined as: Arithmetic mean evaporation reduction rate – Weighted mean evaporation reduction rate = 74.91% − 74.86% = 0.05%.This indicates that the error introduced by the weighted mean calculation method was 0.05%.Although the difference between the weighted average and the arithmetic average in this study is only 0.05%, this discrepancy may translate into a substantial volume of water saved when applied to large water bodies such as reservoirs or irrigation ponds. Furthermore, the weighted average method is theoretically more rigorous, as it is suitable for comparisons across varying climatic conditions and temporal scales, thereby enhancing the generalizability and comparability of the research findings.

#### Confidence interval for evaporation reduction rate.

To evaluate the statistical significance of the weighted average evaporation reduction rate, this study employed the nonparametric Bootstrap method to calculate its 95% confidence interval. The specific steps were as follows: Based on the nine months of monthly observation data (including evaporation from the covered and control groups, the calculated reduction rate, and the weights), repeated random sampling with replacement was performed to construct 10,000 Bootstrap samples. For each sample, the monthly weights were recalculated based on the evaporation from the covered group, and the weighted average reduction rate for that sample was then computed. Finally, the 2.5th and 97.5th percentiles from the empirical distribution of these 10,000 estimated values were taken as the lower and upper bounds of the confidence interval.

Throughout the entire observation period, the weighted average evaporation reduction rate achieved by the hexagonal diamond-shaped floating cover was 74.86%. To quantify the uncertainty of this estimate, the Bootstrap method yielded a 95% confidence interval of (74.34%, 75.38%). This narrow interval indicates that, even when accounting for random monthly observational variation, the cover technology stably delivered an evaporation suppression efficiency of 74.86%, demonstrating high statistical precision in the results.

#### Statistical test of evaporation suppression effect.

**Fig 10:**
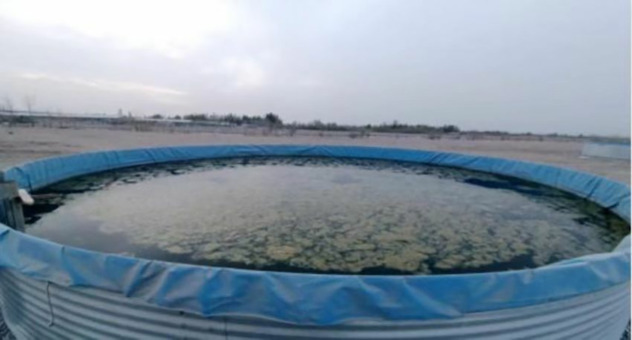
The aquatic ecological conditions of the circular evaporation pool in Group B.

To quantify the evaporation suppression effect of the hexagonal diamond-shaped floating covers and address the statistical inference limitations inherent in a single experimental replicate, this study introduced data from a second completely uncovered control evaporation pond, designated as Group C (Circular Pond 6 in Fig 10). The total evaporation from Group C during the same observation period was 2026.8 mm. Using Group A (covered, 504.4 mm) as the treatment sample and Groups B (2010.7 mm) and C (2026.8 mm) as the control samples, a two-sample t-test (assuming unequal variances) was performed. The statistical results showed that the difference in evaporation between the covered group and the control groups was extremely significant (t (1) = 189.1, p < 0.001). To assess the practical magnitude of the treatment effect, we calculated Cohen’s d effect size, which yielded a value of 133.0. According to the widely adopted criteria in the social sciences (Cohen, 1988), this value indicates that the evaporation suppression effect produced by the floating covers far exceeds the threshold for a “large effect” (d > 0.8), constituting a decisive effect. This provides statistically robust evidence for the effectiveness of the technology. These preliminary findings would benefit from further validation via replicated studies to establish their broader applicability.

### Analysis of the influence mechanism of dust accumulation on evaporation suppression performance and maintenance strategies

The observational data clearly reveal the negative impact of dust accumulation on evaporation suppression efficiency. A single severe sandstorm (May 1, 2024) caused the daily evaporation suppression efficiency to decrease by 4.94% compared to dust-free conditions. More importantly, long-term monitoring indicated a clear gradual degradation trend: in the absence of any cleaning or maintenance, the monthly average evaporation suppression efficiency continuously declined from 76.53% in April 2024 to 73.16% in May 2025, and further decreased to 72.87% in June 2025. Over a period of approximately 14 months, the cumulative reduction amounted to 3.66 percentage points, with no sign of natural recovery observed. The primary physical mechanism involved is the capillary wetting effect: hydrophilic dust particles form “water bridges” in the gaps between the floating covers, substantially increasing the effective evaporation surface area.

#### Performance degradation model and long-term performance curve.

Data spanning two years provide empirical evidence for performance degradation. The trend exhibits two distinct phases:

Short-term event-driven decline: Events such as sandstorms trigger a sharp, partially recoverable drop in efficiency (e.g., the observed 4.94% decrease).

Long-term gradual degradation: Continuous dust accumulation leads to a linear decline in efficiency. Based on data from April 2024 to June 2025, the average monthly degradation rate is approximately 0.26 percentage points. This provides a key parameter for predicting long-term performance: without intervention, the annual efficiency loss in this environment is estimated to be about 3.1 percentage points. This model is validated by continuous observational data.

#### Cleaning frequency requirements and cost/logistics analysis.

Based on the observed annual degradation rate (~3.1%), maintenance planning can be conducted:

Cleaning interval estimation: Assuming an initial efficiency E₀ = 76.5% and a minimum acceptable efficiency E_min_ = 73%, it would take approximately 1.1 years for the efficiency to decline from E₀ to E_min_. Therefore, it is recommended to schedule a comprehensive cleaning annually in similar environments to maintain performance at an acceptable level.

Cost and logistics challenges for large-scale deployment: For large-scale deployments such as reservoirs, manual cleaning is impractical. Considerations must include: the capital investment and operational costs of mechanized cleaning solutions (e.g., specialized cleaning vessels); and logistical challenges such as reservoir management during operations and the treatment of cleaning wastewater.

#### Dust accumulation rates under different seasonal conditions.

Cross-annual data indicate that the degradation rate was not constant. Degradation progressed more rapidly during the spring and summer of 2024 (when dust events were frequent), while the rate was relatively slower from autumn and winter through the early spring of 2025. Seasonal dust phenomena are a key variable affecting the accumulation rate. It is recommended that future studies establish a quantitative model linking dust deposition flux to meteorological parameters to enable more accurate maintenance predictions.

#### Recommendations regarding the impact of dust on evaporation eeduction rate.

Dust accumulation leads to a clear, gradual, and quantifiable degradation in the evaporation suppression efficiency of the floating cover system. The latest data (72.87% as of June 2025) further confirm that, in the absence of maintenance, efficiency will continue to decline to unacceptable levels. Therefore, proactive maintenance is a prerequisite for the sustainable application of this technology. The following recommendations are proposed:

Integrate maintenance into system design: Project feasibility assessments must include long-term performance degradation predictions based on local climate conditions and corresponding maintenance cost analyses.

Develop low-maintenance technologies: Future research should focus on new materials with anti-dust accumulation properties, ease of cleaning, or self-cleaning capabilities to fundamentally reduce maintenance requirements.

Establish standardized monitoring guidelines: Develop simple methods for on-site rapid assessment of dust accumulation levels and system performance status to support scientific maintenance decision-making.

We propose that a practical management strategy could involve scheduling cleaning operations immediately after high-load seasons, such as late spring, to maximize efficiency during peak evaporation periods like summer. Further research should focus on developing surface coatings with low adhesion for floating elements to reduce dust retention.

### Water quality and aquatic ecological impacts

In water quality analysis, Total Dissolved Solids (TDS) refers to the total mass of dissolved inorganic salts (e.g., calcium, magnesium, sodium, potassium ions) and organic matter per unit volume of water, measured in mg/L. TDS concentration reflects the degree of mineralization and pollution characteristics of a water body.

Electrical conductivity is a key indicator of a water body’s ability to conduct electricity, representing the total concentration of dissolved ions and salinity levels. Its value is strongly correlated with temperature, ion species, and concentration. Measurements are typically standardized to 25°C (unit: μS/cm).

pH value is a core indicator of water acidity or alkalinity. It directly influences the stability of aquatic ecosystems and the processes of substance transport and transformation.

As shown in Tables 13–15, the water bodies covered with hexagonal diamond-shaped floaters demonstrated superior water quality parameters compared to the uncovered control group, including lower total dissolved solids (TDS), reduced electrical conductivity, and a more favorable PH.

**Table 13:** The total amount of dissolved solids in the water bodies of the two groups of evaporation pools (Unit: mg*L^-1^).

Date (Month/Day)	The total amount of dissolved solids in the water of Group A circular evaporation pool (mg*L^-1^)	The total amount of dissolved solids in the water of Group B circular evaporation pool
5/1	822	979
5/11	832	1003
5/21	845	1067
5/31	883	1125

**Table 14:** The electrical conductivity of the water bodies in the two groups of evaporation ponds (Unit: μs*cm^-1^).

Date (Month/Day)	The conductivity of the water body in the circular evaporation pool of Group A	The conductivity of the water body in the circular evaporation pool of Group B
5/1	1534	1958
5/11	1664	2126
5/21	1690	2160
5/31	1766	2286

**Table 15:** The PH of the water in the two groups of oevapration tanks.

Date (Month/Day)	PH value of the circular evaporation pool in Group A	PH value of the circular evaporation pool in Group B
5/1	8.39	9.34
5/11	8.46	9.37
5/21	8.54	9.49
5/31	8.51	9.66

#### Mechanism of water quality modulation by floating covers.

The significant improvements in water quality parameters under the HDFCs are direct hydrological and geochemical consequences of evaporation suppression. Mitigation of Salinization (TDS & EC): Evaporation removes pure water, concentrating all dissolved salts and ions-—a primary driver of salinization in arid regions. By inhibiting approximately 75% of evaporation (Table 4, May), the covers drastically reduced the net water loss, thereby slowing the concentrative process and resulting in significantly lower end-of-month TDS and EC values compared to the uncovered control.

Modulation of pH and Alkalinity: The substantially higher pH in the uncovered group (B: ~ 9.5; A: ~ 8.5) is attributed to evaporative concentration of the carbonate system. In arid region waters, alkalinity is dominated by bicarbonate. Intensive evaporation increases the concentrations of all ions, shifting the carbonate equilibrium towards carbonate formation. The hydrolysis of produces hydroxide ions, elevating the pH to strongly alkaline levels. The covers curtailed this alkalinity concentration process, maintaining a pH closer to initial, more neutral conditions-a crucial co-benefit for agricultural water use.

#### Water quality analysis and statistical significance.

Key water quality parameters were monitored during May, a month of relatively high evaporative demand, to preliminarily assess the secondary benefits of the covers (Tables 8–10). Paired-sample t-tests (n = 4 paired dates) were conducted to evaluate the significance of differences between the covered (Group A) and uncovered (Group B) reservoirs. The tests confirmed highly statistically significant differences for all parameters: Total Dissolved Solids (TDS: mean difference = 198.0 mg/L, t(3) = 9.78, p = 0.001), Electrical Conductivity (EC: mean difference = 469.0μS/cm, t(3) = 23.76, p < 0.001), and pH (mean difference = 0.99, t(3) = 18.28, p < 0.001). This indicates that the covers significantly mitigated the increase in solute concentration and pH under intense evaporation.

#### Aquatic ecological impacts.

**Fig 11:**
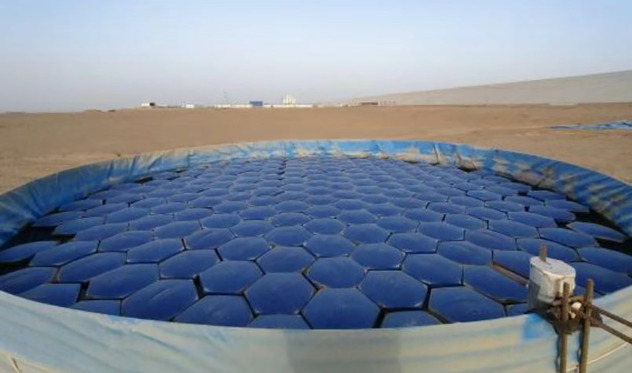
The aquatic ecological conditions of the circular evaporation pool in Group A.

From April onwards, extensive algal growth was observed in the uncovered circular evaporation pond (Group B), as depicted in Fig 10. In contrast, minimal algal growth occurred in the covered pond (Group A) during the same period (Fig 11). Excessive algal proliferation poses significant risks to water quality. Driven by eutrophication, algal blooms undergo substantial diel respiration fluctuations, causing dissolved oxygen concentrations to swing dramatically from supersaturation during daylight to severe hypoxia at night. This can lead to suffocation and mortality of aquatic organisms such as fish.

Furthermore, the decomposition of dead algal biomass consumes substantial oxygen and releases toxic compounds, including hydrogen sulfide and ammonia, further degrading water quality. Algal mats also block sunlight, inhibiting the growth of submerged macrophytes and disrupting aquatic ecological balance, while increasing water treatment costs. This process establishes a detrimental cycle of “algal proliferation → water quality deterioration → ecological collapse,” severely compromising water body functionality and socio-economic sustainability. Thus, the aquatic ecosystem under the coverage of hexagonal diamond-shaped floaters maintained a healthier state.

### Cost analysis for deployment in large evaporation pond 1

#### Life cycle cost of HDFCs.

The pond area is 1055 m² (Fig 6), and a total of 6812 hexagonal diamond-shaped floating covers were installed. Each floating cover costs 23 Chinese Yuan (CNY), resulting in a total material cost of 156,776 CNY.

Installation labor: Typically accounts for 10%–20% of the material cost, covering labor and machinery (e.g., boats). (Estimated at 15% of the material cost): 23,500 CNY.

Maintenance and cleaning: Annual inspection and dust removal: 2,000 CNY per year.

Storage, monitoring, and management during non-use periods: Excess floating covers are stored in the reservoir management station warehouse: 1,000 CNY per year.

Replacement of damaged units: The annual damage rate is estimated at 1%–3%. Assuming a 2% rate, the annual replacement quantity is calculated as 6812 × 0.02 = 136.24 units. The replacement cost is 136.24 × 23 = 3,133.52 CNY per year.

Monitoring costs: Regular monitoring of water quality in the covered area and the integrity of the floating covers: 1,500 CNY per year.

Total annual operating cost = 2,000 + 1,000 + 1,500 + 3,133.52 = 7,633.52 CNY per year.

Recycling value: Each floating cover weighs 1.8 kg, with a total weight of 6812 × 1.8 = 12,261.6 kg = 12.2616 metric tons.

At a recycling price of 7,000 CNY per ton, the total recycling value is 12.2616 × 7,000 = 85,831.2 CNY (realized at the end of the 20th year).

Total life cycle cost

Total cost = Initial investment + Total operating costs over 20 years – Recycling value

= 180,276 + 152,670.4–85,831.2 = 247,115.2 CNY.

The estimated total life cycle cost over 20 years is approximately 247,115 CNY.

#### Return on investment calculation.

According to the Review Opinions on Implementing the Strictest Water Resources Management System and Enforcing the “Three Red Lines” Control Indicators in Various Counties and Cities of the Hotan Region and the 14th Division of the Xinjiang Production and Construction Corps, the total annual water consumption in the project area is 118.64 million m³. Statistics on water usage in the project area are shown in Table 16.

**Table 16:** Water Consumption Statistics.

Water Use Category	Annual Water Consumption (10,000 m³)	Proportion of Water Use
Agricultural Water Use	7532	0.63
Domestic Water Use	2090	0.18
Industrial Water Use	1950	0.16
Other Water Use	180	0.02
Total Water Use	11864	1.00

The local water prices for agricultural, domestic, industrial, and other uses are known to be 0.2 CNY/m³, 2 CNY/m³, 3 CNY/m³, and 1.5 CNY/m³, respectively. Based on the proportion of water usage for each category, the average water price is calculated to be 0.996 CNY/m³. The evaporation reduction for the evaporation pond from June to September is 857.71 m³. The reduction in evaporation for the circular evaporation pond during the same period (845.7 mm) accounts for 56.14% of the total evaporation reduction during the non-freezing period (1506.3 mm). Therefore, the evaporation reduction during the non-freezing period is 1527.8 m³. The annual evaporation reduction is approximated to be 1550 m³. Assuming a 20-year operational lifespan, the direct economic benefit from water savings for this evaporation pond amounts to 30,876 CNY. Considering only the profit from water savings and the comprehensive water price, the project does not achieve positive financial growth within the service life of the covering material.

The economic analysis of this study reveals that under the current low water price (approximately 0.99 CNY/m³), the direct water-saving revenue from this covering technology cannot cover its full life-cycle costs. This highlights a typical dilemma in water resource management in arid regions: a severe structural imbalance exists between the cost of advanced water-saving technologies and the prevailing market price of water as a commodity.

Therefore, this technology should not be viewed merely as a commercial investment project. Instead, it should be positioned as “water security infrastructure” that safeguards regional water security. Its core value lies in directly reducing inefficient water loss through engineering measures, thereby enhancing the resilience of the water supply system. Its benefits extend broadly to social, ecological, and strategic dimensions, which are difficult to fully monetize. Consequently, the large-scale deployment of this technology depends on two conditions: first, addressing the cost gap through water price adjustments or subsidy mechanisms; and second, prioritizing its application in areas of extreme water scarcity or in contexts where water holds higher economic value (e.g., urban water supply or high-value agricultural zones), in order to balance technological effectiveness with economic feasibility.

## Conclusion

This study was conducted in the arid southern region of Xinjiang, utilizing three circular evaporation ponds with consistent structural parameters (diameter: 6 m, height: 2.3 m). Hexagonal diamond-shaped floating covers were selected as the water-surface covering material, and evaporation suppression experiments were carried out using the floating coverage method. The experimental setup included one treatment pond covered with the floating covers and two uncovered control ponds. Evaporation experiments were performed under static water conditions during the non-freezing period of 2024 (March 19 to November 30), supplemented by dynamic water environment validation tests from June to September of the same year in a large, unshielded evaporation pond covering 1055 m². Based on the comprehensive study, the main conclusions are as follows:

(1) Under static water conditions, the HDFCs achieved a weighted average evaporation suppression rate of 74.86% (95% CI: 74.34%–75.38%). Statistical tests indicated an extremely significant difference between the covered and control groups (p < 0.001, Cohen’s d = 133.0). In dynamic water environments, the suppression rate remained above 70%, demonstrating the potential of this technology for application in practical open-water bodies such as reservoirs.
(2) Dust accumulation is the primary factor leading to the gradual decline in evaporation suppression efficiency. Under long-term operation without maintenance, efficiency is expected to decrease by approximately 3.1 percentage points annually. It is recommended to adopt proactive maintenance strategies, such as annual cleaning at the end of late spring, to maintain high efficiency during the peak summer evaporation period. Future research should focus on developing low-adhesion surface coatings to reduce maintenance requirements.
(3) Based on measured data and the assumption of linear proportionality, if 98% coverage is achieved under ideal geometric conditions, the theoretical evaporation suppression rate could reach 88.59%.
(4) Life cycle analysis showed that the water footprint of producing the hexagonal diamond-shaped floating covers (approximately 35.24 m³) could be fully recovered through water-saving benefits within about 0.83 non-freezing periods (7 months) of operation. The carbon emission reduction benefits from water savings could offset approximately 74% of the initial production carbon cost. The floating covers are made of high-density polyethylene (HDPE), which is highly recyclable at the end of its service life, further reducing the environmental footprint over the full life cycle.
(5) Through over a decade of field validation in extreme environments, the HDPE material used in this study demonstrated excellent durability. The addition of appropriate amounts of UV absorbers, light stabilizers, and antioxidants to the HDFCs effectively delayed the aging process, with an expected service life of up to 20 years. Future attention should be given to the potential environmental impacts of long-term aging and microplastic release.
(6) While suppressing evaporation, the HDFCs also provided significant co-benefits for water quality and ecological health. The covers effectively mitigated the increase in Total Dissolved Solids (TDS) and Electrical Conductivity (EC), stabilized the pH at a level closer to neutral (covered group: ~ 8.5; uncovered group: ~ 9.5), with all differences being highly statistically significant (p < 0.001). Additionally, the covers effectively inhibited excessive algal growth.
(7) Economic analysis revealed that, under the current water price (approximately 0.99 CNY/m³), the direct water-saving revenue alone is insufficient to cover the full life cycle costs. This technology is more appropriately positioned as a “water security infrastructure” to safeguard regional water security. Its large-scale application depends on adjustments to water pricing policies, subsidy mechanisms, or prioritized deployment in high-value water-use scenarios.
(8) The technology has the potential for year-round application. Theoretical analysis suggests that during the freezing period, it can still function by suppressing ice sublimation, and the annual evaporation suppression rate is expected to remain above 74.86%.

In summary, the hexagonal diamond-shaped floating cover technology is an efficient, environmentally friendly, and engineering-reliable solution with promising application prospects for evaporation control and aquatic ecosystem maintenance in arid region reservoirs.
